# Dysregulation of miR-6868-5p/FOXM1 circuit contributes to colorectal cancer angiogenesis

**DOI:** 10.1186/s13046-018-0970-5

**Published:** 2018-11-28

**Authors:** Ye Wang, Meijuan Wu, Zengjie Lei, Mengxi Huang, Zhiping Li, Liya Wang, Qijun Cao, Dong Han, Yue Chang, Yanyan Chen, Xiaobei Liu, Lijun Xue, Xiaobei Mao, Jian Geng, Yanan Chen, Tingting Dai, Lili Ren, Qian Wang, Hongju Yu, Cheng Chen, Xiaoyuan Chu

**Affiliations:** 1 Departments of Medical Oncology, Jinling Hospital, Nanjing Clinical School of Southern Medical University, Nanjing, Jiangsu Province China; 2 Department of Medical Oncology, Jinling Hospital, School of Medicine, Nanjing University, Nanjing, Jiangsu Province China

**Keywords:** Colorectal cancer, FOXM1, miRNA, Angiogenesis

## Abstract

**Background:**

Transcription factor forkhead box M1 (FOXM1) is a crucial regulator in colorectal cancer (CRC) progression. However, the regulatory mechanisms causing dysregulation of FOXM1 in CRC remain unclear.

**Methods:**

Dual-luciferase reporter assay was conducted to determine FOXM1 as miR-6868-5p target. The function of miR-6868-5p and FOXM1 in CRC angiogenesis was verified in vitro. Intratumoral injection model was constructed to explore the effect of miR-6868-5p on angiogenesis in vivo. Chromatin immunoprecipitation assays were used to assess direct binding of H3K27me3 to the miR-6868 promoter.

**Results:**

Through integrated analysis, we identified miR-6868-5p as the potent regulator of FOXM1. Overexpression of miR-6868-5p in CRC cells inhibited the angiogenic properties of co-cultured endothelial cells, whereas silencing of miR-6868-5p had opposite effects. In vivo delivery of miR-6868-5p blocked tumor angiogenesis in nude mice, resulting in tumor growth inhibition. Rescue of FOXM1 reversed the effect of miR-6868-5p on tumor angiogenesis. Further mechanistic study revealed that FOXM1 promoted the production of IL-8, which was responsible for the miR-6868-5p/FOXM1 axis-regulated angiogenesis. Reciprocally, FOXM1 inhibited miR-6868-5p expression through EZH2-mediated H3K27me3 on miR-6868-5p promoter, thus forming a feedback circuit. Clinically, the level of miR-6868-5p was downregulated in CRC tissues and inversely correlated with microvessel density as well as levels of FOXM1 and IL-8 in tumor specimens.

**Conclusions:**

Together, these data identify miR-6868-5p as a novel determinant of FOXM1 expression and establish a miR-6868-5p/FOXM1 regulatory circuit for CRC angiogenesis, providing potential target for CRC treatment.

**Electronic supplementary material:**

The online version of this article (10.1186/s13046-018-0970-5) contains supplementary material, which is available to authorized users.

## Introduction

Colorectal cancer (CRC) is the third most common cancer and the third leading cause of cancer-related death in the world [1]. In recent years, the incidence and mortality of CRC has increased rapidly in China [2]. The development and progression of CRC is regulated by various genetic and epigenetic manners. It is imperative to understand the molecular mechanisms of CRC progression and identify novel targets for CRC therapy.

Forkhead box M1 (FOXM1) is a transcription factor that belongs to the FOX superfamily, characterized by a conserved winged helix DNA-binding domain [3]. FOXM1 is frequently overexpressed in a variety of cancers, including CRC. FOXM1 exhibits potent oncogenic properties in promoting cell proliferation, chromosome instability, stem cell self-renewal, and functions as activator of tumor metastasis through enhancing epithelial–mesenchymal transition, cell migration, invasion and angiogenesis. Indeed, our previous studies have shown that FOXM1 expression predicted poor prognosis of CRC patients, and FOXM1 promoted the metastasis and chemoresistance of CRC cells [4–7]. However, little is known about the underlying mechanisms responsible for the elevated FOXM1 expression in CRC.

MicroRNAs (miRNAs) are important post transcriptional regulators, which negatively regulate gene expression by targeting the 3′-untranslated region (3’-UTR) of mRNAs [8]. Over the past decades, studies have revealed that miRNA deregulation is implicated in tumor development and progression, such as tumor growth, metastasis and immune responses. Recent advancement in miRNA-based therapy has made it a promising way for cancer treatment [9–12]. Therefore, identification of critical miRNAs involved in CRC progression could help us to develop better therapeutic strategies.

In this study, we identified miR-6868-5p, whose expression was downregulated in CRC, as a potential mechanism for increased FOXM1 expression in CRC. We reported that miR-6868-5p suppressed tumor angiogenesis by inhibiting FOXM1. In turn, FOXM1 inhibited miR-6868-5p expression through promoter histone methylation. Our results indicate a novel miR-6868-5p/FOXM1 feedback loop as determinant of CRC angiogenesis, and provide promising therapeutic targets for CRC.

## Methods

### Cells and cell transfection

Human colon cancer cell lines HCT8 and HCT116 were maintained in RPMI 1640 medium supplemented with 10% fetal bovine serum (FBS, GIBCO) and 1% Penicillin-Streptomycin Solution (HyClone). Human umbilical vein endothelial cells (HUVECs) were cultured in DMEM medium with 10% FBS and 1% Penicillin-Streptomycin Solution. The colon cancer cells were plated in 6-well plate and cultured for 24 h, and then miRNA mimic/inhibitor and (or) FOXM1 plasmid were transfected into cells using GenMute™ siRNA & DNA Transfection Reagent (SignaGen). Twelve hours post transfection, cells were washed in PBS and added with 2 ml fresh medium.

### HUVEC proliferation, scratch and tube formation assays

After tumor cells transfected with miRNA mimic/inhibitor and (or) FOXM1 plasmid for 48 h, the conditioned medium (CM) was collected and centrifuged at 2000 rpm for 10 min to remove debris, and used for HUVEC proliferation, scratch and tube formation assays. For proliferation assay, HUVECs were seeded in 96-well plate (2.5 × 10^3^ cells per well). Twenty-four hours later, the medium were removed and 200 μl CM per well were added, and Cell Counting Kit-8 was used to detect cell proliferation.

For Scratch assay, HUVEC were seeded at 2.0 × 10^5^ cells per well in 6-well plate. When cells reached about 90% confluence, a 200 μl tip was used to scratch the cells. The cells were then washed with PBS twice and incubated with 2 ml CM per well. The scratch was photographed at 0 h, 12 h and 24 h time points under microscope. The migration distance was determined using Image Pro Plus software.

For tube formation assay, 15 μ-Slide Angiogenesis plates (ibidi) were covered with 10 μl/well of Matrigel (Corning) and incubated at 37 °C for 35 min for hardening. HUVEC (1.0 × 10^4^/well) resuspended in CM were plated onto the pre-coated plate and incubated at 37 °C for 4-6 h.The IL-8 neutralizing antibody (5 μg/ml, R&D systems) was added to the CM in the rescue experiments. Then the capillary-like structures were photographed and the number of nodes was quantified using Image Pro Plus software.

### HUVEC transwell migration assay

Colon cancer cell lines HCT8 and HCT116 were cocultured with HUVEC by using 24-well cell migration chambers with 8 μm pore size inserts (Corning). First, HCT8 and HCT116 were plated in the lower chamber and transfected with miRNAs mimic/inhibitor and (or) FOXM1 plasmid for 12 h, and then cultured in 2% FBS medium. HUVEC (1.25 × 10^4^/well) were seeded into the insert and cocultured with colon cells for 24 h. Non-migrated HUVEC were removed by cotton swabs from the upper surface of the insert and cells on the lower surface of the insert were fixed and stained. Migrated cells were photographed by digital microscope in five random fields and number of migrated cells was calculated and determined as the mean value.

### Luciferase reporter assay

The fragment of the FOXM1 3’UTR containing the miR-6868-5p targeting sequence was cloned into the pmirGLO dual Luciferase reporter plasmid, which was designed by Genomeditech (Shanghai, China). For the reporter assay, HCT116 were seeded at 48-well plate and co-transfected with miR-6868-5p mimics/inhibitor and pmirGLO-FOXM1–3’UTR-WT (wild type) or MT (mutant) constructs. Each group was run in triplicate in 48-well plates. The luciferase activity was detected by Dual-Luciferase Reporter Assay System (Promega) after 36 h of transfection. Firefly luciferase activity was normalized against Renilla luciferase activity.

### Quantitative real-time (qRT)-PCR

Total RNA was extracted from cells using TRIzol reagent (Invitrogen). For mRNA, cDNA was synthesized by using PrimeScript™ RT reagent Kit (Takara) according to the manufacturer’s instructions. qRT-PCR was performed using SYBR Green (Takara) with β-actin as a house keeping control. For miRNA, miDETECT A Track™ miRNA qRT-PCR Starter Kit (RiboBio) was used to synthesize cDNA and analysis the expression levels of miRNA. The sequences of primers were listed in Additional file 1: Table S2.

### Western blot

Cells were lysed by RIPA buffer containing protease inhibitor cocktail (MedChem Express). Protein concentrations were determined by BCA Protein Assay Kit (KeyGENBioTECH). Equal amount of protein was loaded and separated by SDS–PAGE, and transferred onto PVDF membranes (Bio-Rad Laboratories). Membranes were incubated with primary antibodies against FOXM1 (1:100, Santa Cruz), β-actin (1:2000, Cell Signaling Technology) and H3K27me3 (1:1000, Abcam) overnight at 4 °C, followed by incubation with horseradish peroxidase-conjugated secondary antibodies at room temperature for 1 h. The ChemiDoc MP system was employed to detect the protein signals via chemiluminescent substrate ECL kit (Merck Millipore).

### Chromatin immunoprecipitation (ChIP) assay

The chromatin immunoprecipitation assay was performed using Magna ChIP HiSens Kit (Millipore) according to the manufacturer’s instructions. Chromatin was immunoprecipitated with FOXM1 or H3K27m3 (Abcam) antibody and analyzed using qRT-PCR. The sequences of primers for ChIP assay were listed in Additional file 1: Table S3.

### ELISA assay

IL-8 protein secreted from cells was quantified by Human IL-8 ELISA kit (Dakewe) according to the manufacturer’s protocol.

### Mouse xenograft model

HCT116 were resuspended at 3.5 × 10^6^ cells/ml and injected (0.1 ml per mice) subcutaneously into athymic nude mice (5-week-old, male). One week after inoculation, tumor-bearing mice were randomly divided into two groups, and agomiR-NC and agomiR-6868-5p (RiboBio) were injected at multipoints into respective xenografts. The agomiR (20 nM) were injected every 3 days. Tumor volumes were measured by caliper and calculated using formula: length×width^2^/2. After the injection of 6 times, mice were euthanized and tumors were surgically dissected, followed with histopathologic examination. Animal maintenance and experimental procedures were approved by the Institutional Animal Care and Use Committee of Jinling Hospital.

### Immunohistochemistry and immunofluorescence

Paraffin embedded sections were deparaffinized, rehydrated, followed by antigen retrieval. For immunohistochemistry staining, the sections were incubated with primary antibodies against CD31 (1:15, abcam), FOXM1 (1:100, Santa Cruz), or IL-8 (1:200, Servicebio), followed with horseradish peroxidase-conjugated secondary antibody. The slides were finally incubated with diaminobenzidine (DAB) (Dako) and counterstained with hematoxylin. For immunofluorescence analysis, the sections were co-stained with mouse anti-CD31(1:25, abcam) and rabbit anti-NG-2(1:100, R&G Systems) primary antibodies, followed by incubation with Alexa Fluor555-conjugated anti-mouse-IgG and Alexa Fluor 488-conjugated anti-rabbit-IgG. Nuclear staining was conducted using DAPI. Representative images were acquired using an Olympus IX70 microscope.

### Gene ontology enrichment analysis

The target genes of miR-6868-5p were predicted using two bio-informatic prediction tools (i.e TargetScan and miRDB), and genes co-targeted by both prediction tools were subjected to Gene Ontology (GO) enrichment analysis using the ‘clusterProfiler’ package in R. The ‘clusterProfiler’ estimates the significance of enrichment for a collection of genes, and hypergeometric distribution model was used to calculate a *P* value for GO categories and q-values were also calculated for FDR control.

### Statistical analysis

Data were analyzed employing Excel software and SPSS2.0. Data were presented as mean ± SD. Differences between groups were analyzed by Student’s t test or ANOVA. Pearson correlation analysis was conducted to assess the correlation between two variables. *P* < 0.05 was considered statistically significant.

## Results

### FOXM1 is negatively regulated by miR-6868-5p

**Fig. 1:**
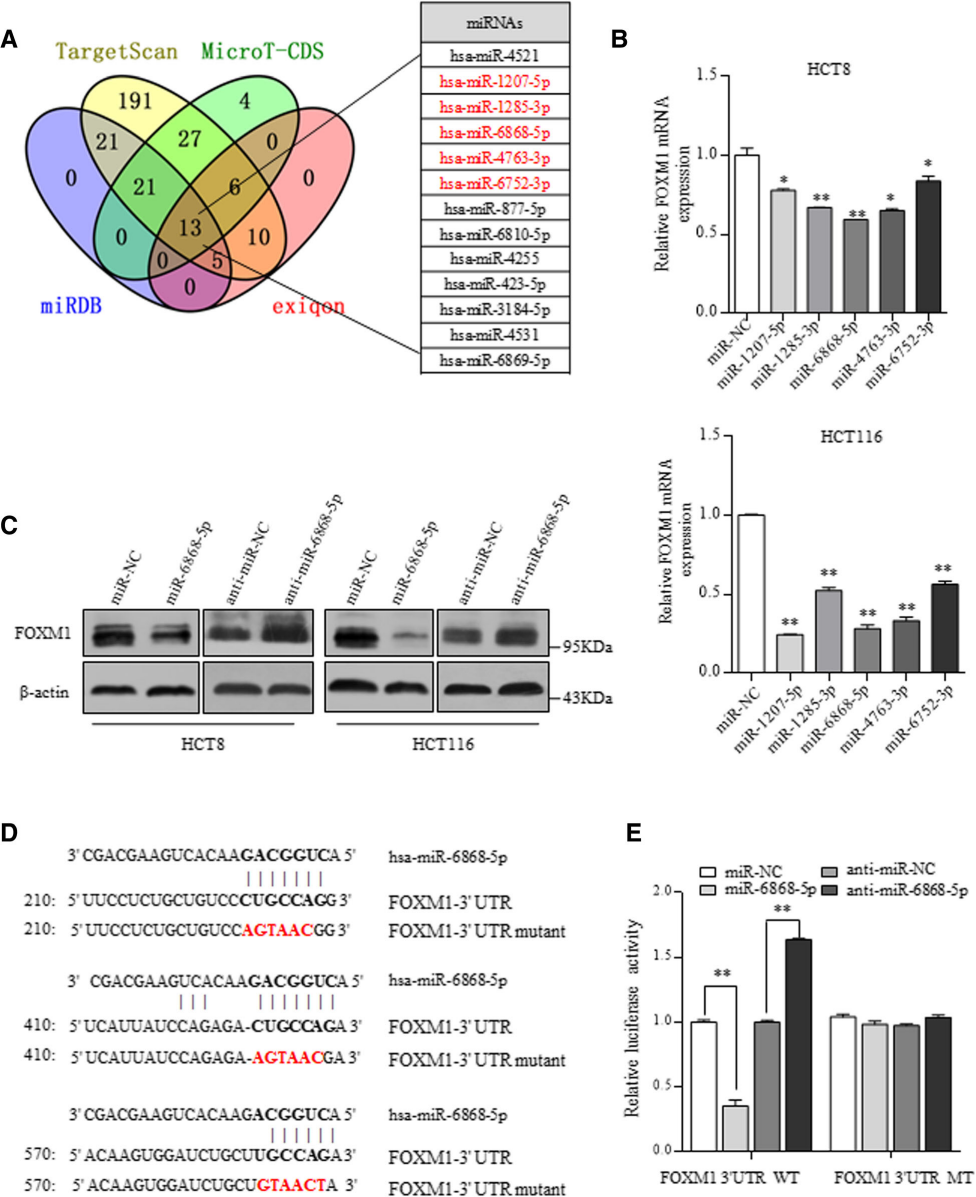
FOXM1 is negatively regulated by miR-6868-5p. **a** FOXM1 3’-UTR region was used for analysis of miRNA binding sites by using four different bioinformatic tools. miRNAs with at least 3 putative binding sites in at least two prediction tools were marked in red. **b** qRT-PCR analysis of FOXM1 expression in HCT8 and HCT116 cells transfected with indicated miRNA mimics. **c** Western blot analysis of FOXM1 expression in HCT8 and HCT116 cells transfected with miR-6868-5p mimic or miR-6868-5p inhibitor. **d** Locations of miR-6868-5p binding sites in the FOXM1 3’-UTR and the mutated FOXM1 3’-UTR are shown. **e** Luciferase activity of the wild-type (WT) or mutant (MT) FOXM1 3’-UTR reporter was measured in HCT116 cells transfected with miR-6868-5p mimic or inhibitor. *p < 0.05, **p < 0.01

To explore potential miRNAs in the regulation of FOXM1, we used four different bio-informatic prediction tools to analyze if miRNA-binding sites are present in the FOXM1 3’-UTR region and identified 13 miRNAs from intersection (Fig. 1a). We screened out 5 miRNAs with at least 3 putative binding sites in the 3’-UTR of FOXM1 in at least two of the four prediction tools (Additional file 1: Table S1). PCR validation showed that overexpression of miR-6868-5p most significantly reduced RNA levels of FOXM1 in HCT8 and HCT116 cells (Fig. 1b). By detecting the endogenous expression of miR-6868-5p and three other miRNAs that have been reported to function in CRC cells, we found that miR-6868-5p level was comparable to the other three miRNAs (Additional file 1: Figure S1A). Therefore, the endogenous level of miR-6868-5p was high enough to make its knockdown effect convincing. Upon overexpressing miR-6868-5p, the protein level of FOXM1 was decreased, while anti-miR-6868-5p inhibitor increased the FOXM1 protein level (Fig. 1c and Additional file 1: Figure S1B-C). To evaluate the function of putative binding sites of miR-6868-5p in FOXM1 3’-UTR, we inserted the wild-type (WT) or binding sites-mutated (MT) FOXM1 3’-UTR region into a reporter construct (Fig. 1d). Overexpression of miR-6868-5p significantly reduced reporter activity in the FOXM1 3’-UTR-WT construct. However, the inhibitory effect exerted by miR-6868-5p was abrogated when the binding sites were mutated (Fig. 1e). Consistently, the relative luciferase activity was enhanced by anti-miR-6868-5p inhibitor, while mutation of the predicted binding sites blunted the effect (Fig. 1e). These results indicate that FOXM1 is a direct downstream target of miR-6868-5p in CRC cells.

### miR-6868-5p inhibits CRC angiogenesis by targeting FOXM1

**Fig. 2:**
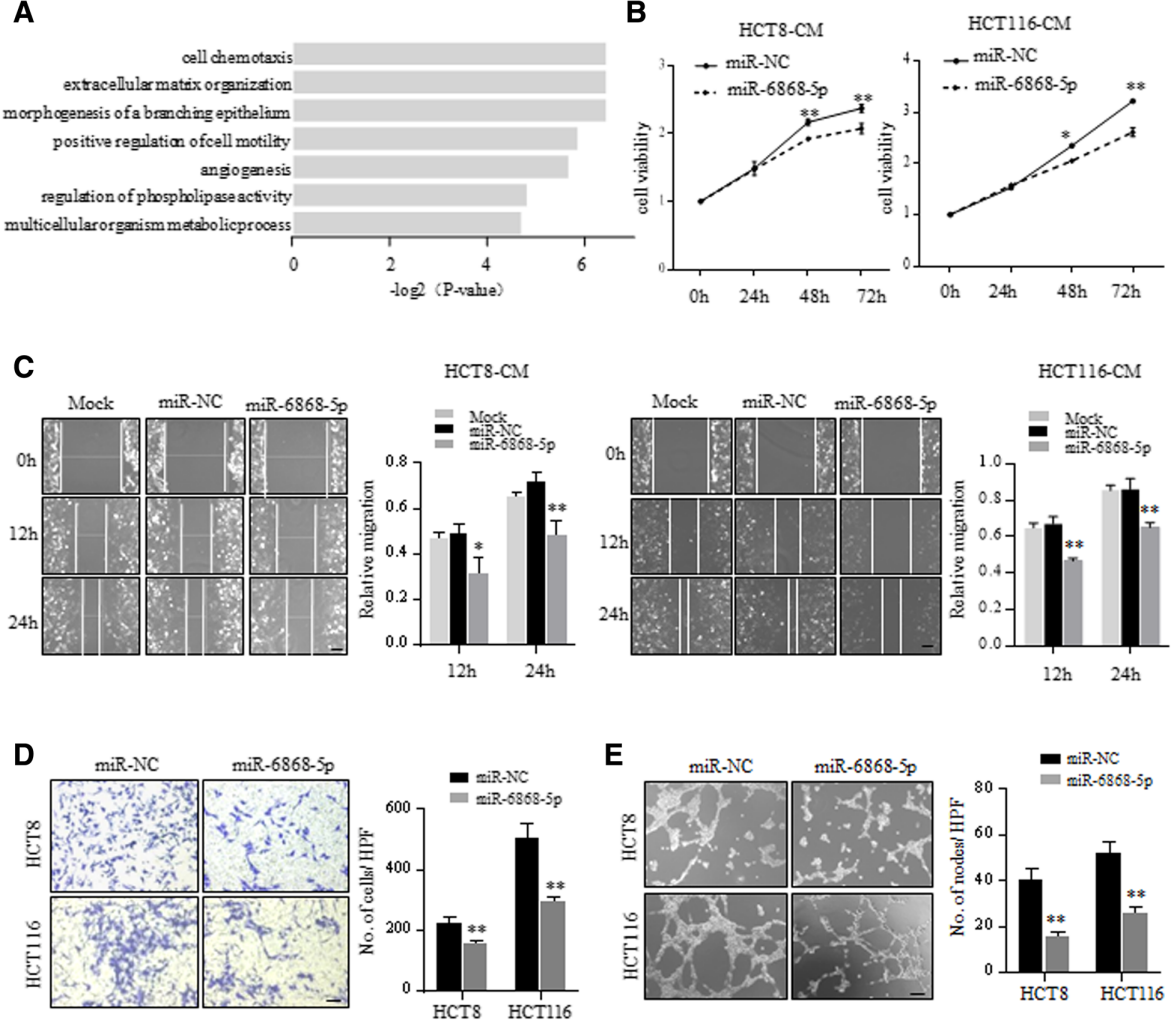
Overexpression of miR-6868-5p inhibits CRC angiogenesis. **a** Gene ontology of miR-6868-5p-targeted gene expression events. Fisher exact *p* values were plotted for each enriched functional category. **b** and **c** HUVECs were treated with the CM from HCT8 or HCT116 cells transfected with indicated miRNA mimics. Cell viability (**b**) and migration ability (**c**) of HUVECs were measured by CCK8 assay and wound healing assay respectively. Scale bar = 20 μm. **d** HUVECs were co-cultured with HCT8 or HCT116 cells transfected with indicated miRNA mimics in transwell apparatus with 8 μm pore size. Migrated HUVECs were quantified after co-culture for 24 h. Scale bar = 20 μm. **e** HUVECs were treated with the CM from indicated cells, and subjected to tube formation assay. Scale bar = 20 μm. *p < 0.05, **p < 0.01

**Fig. 3:**
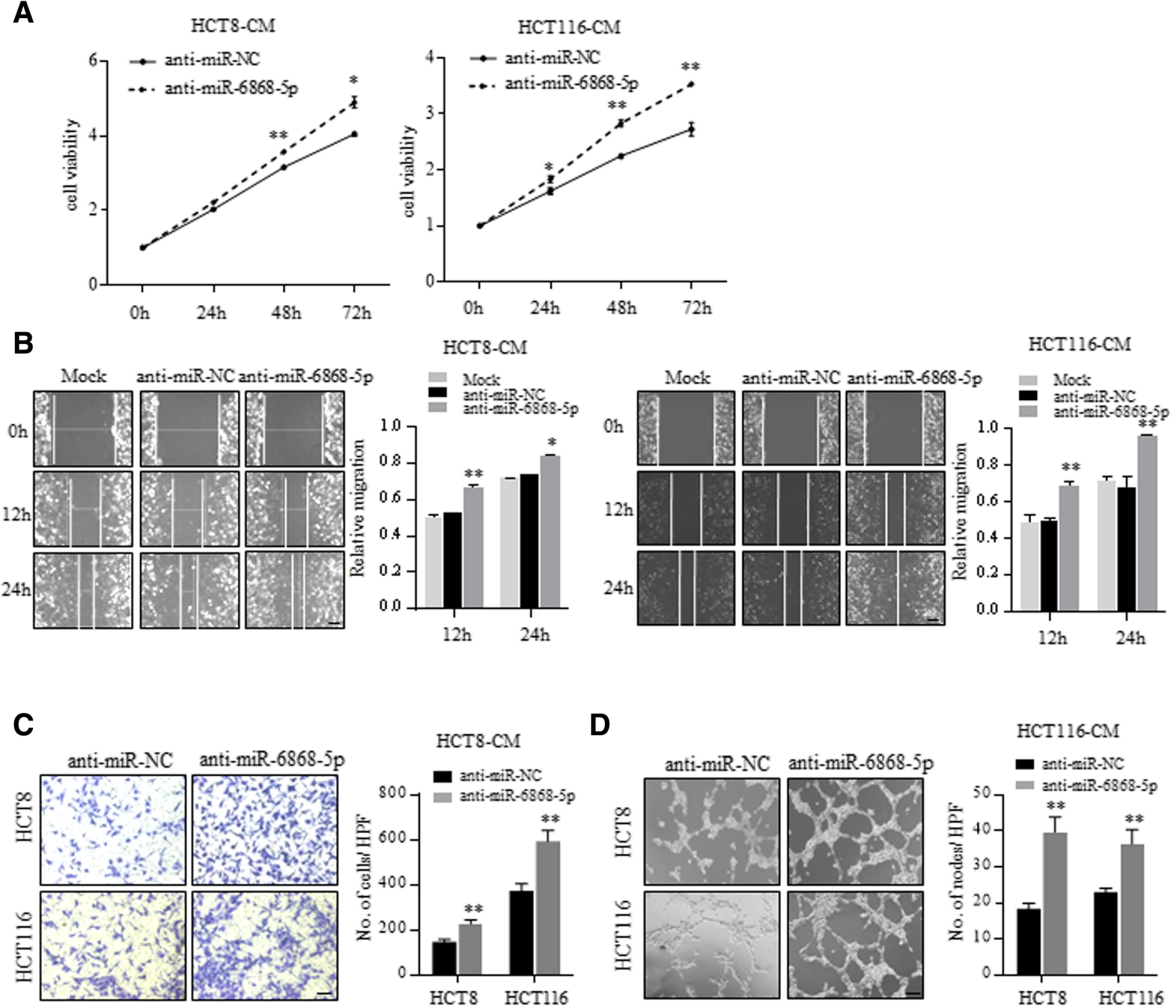
Inhibition of miR-6868-5p promotes CRC angiogenesis. **a** and **b** HUVECs were treated with the CM from HCT8 or HCT116 cells transfected with indicated miRNA inhibitors. Cell viability (**a**) and migration ability (**b**) of HUVECs were measured by CCK8 assay and wound healing assay respectively. Scale bar = 20 μm. **c** HUVECs were co-cultured with HCT8 or HCT116 cells transfected with indicated miRNA inhibitors in transwell apparatus. Migrated HUVECs were quantified after co-culture for 24 h. Scale bar = 20 μm. **d** HUVECs were treated with the CM from indicated cells, and subjected to tube formation assay. Scale bar = 20 μm.*p < 0.05, **p < 0.01

Gene ontology analysis of downstream target genes of miR-6868-5p revealed that angiogenesis was significantly enriched (Fig. 2a). Since FOXM1 is known to promote tumor angiogenesis [13–15] and proved as the direct target of miR-6868-5p, we reasoned that miR-6868-5p may regulate tumor angiogenesis through modulating FOXM1 expression. To test his hypothesis, we first determined the effect of miR-6868-5p on tumor angiogenesis. We exposed conditioned medium (CM) from CRC cells to human umbilical vascular endothelial cells (HUVECs). Compared with CM from control CRC cells, CM from miR-6868-5p overexpressing CRC cells inhibited the proliferation (Fig. 2b) and migration (Fig. 2c and d) of HUVECs. Moreover, forced expression of miR-6868-5p resulted in significant reduction in endothelial tube formation (Fig. 2e). The effect of miR-6868-5p on CRC angiogenesis was further confirmed by inhibiting miR-6868-5p expression. As compared with the CM from control group, CM from miR-6868-5p inhibitor group promoted the proliferation (Fig. 3a), migration (Fig. 3b and c) and tube formation (Fig. 3d) of HUVECs. These data indicated that miR-6868-5p could suppress CRC angiogenesis.

**Fig. 4:**
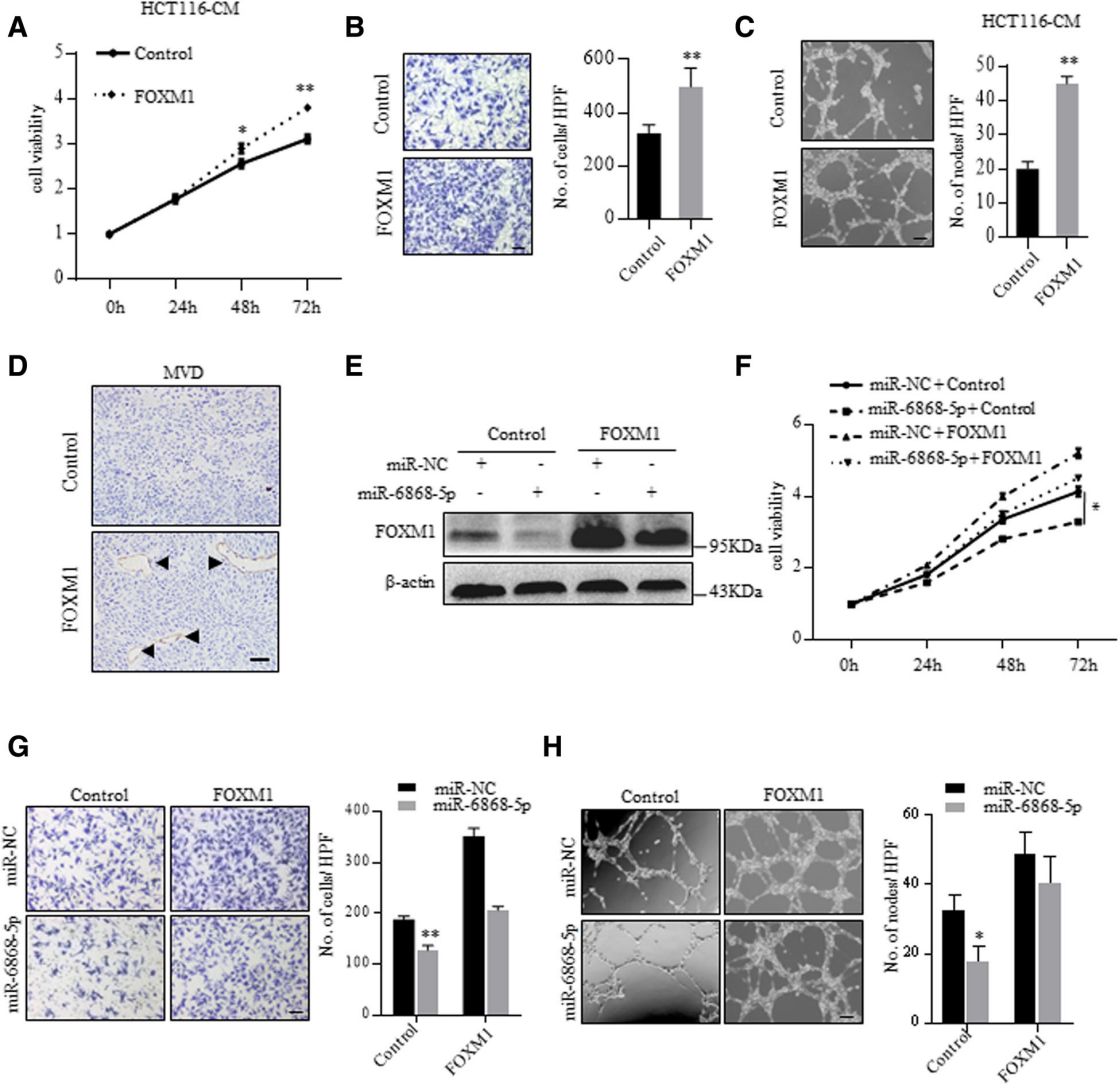
miR-6868-5p inhibits CRC angiogenesis by targeting FOXM1. **a** HUVECs were treated with the CM from HCT116 cells transfected with indicated vectors. Cell viability of HUVECs was measured by CCK8 assay. **b** HUVECs were co-cultured with HCT116 cells transfected with indicated vectors in transwell apparatus. Migrated HUVECs were quantified after co-culture for 24 h. Scale bar = 20 μm. **c** HUVECs were treated with the CM from indicated cells, and subjected to tube formation assay. **d** Representative images of IHC staining for CD31 in control and FOXM1 overexpressing tumors. Scale bar = 20 μm. **e** Western blot analysis of FOXM1 expression in indicated HCT116 cells. **f** HUVECs were treated with the CM from HCT116 cells transfected with indicated miRNA mimics and vectors. Cell viability of HUVECs was measured by CCK8 assay. **g** HUVECs were co-cultured with HCT116 cells transfected with indicated miRNA mimics and vectors in transwell apparatus. Migrated HUVECs were quantified after co-culture for 24 h. Scale bar = 20 μm. **h** HUVECs were treated with the CM from indicated cells, and subjected to tube formation assay. Scale bar = 20 μm. *p < 0.05, **p < 0.01

Consistent with previous reports, CM from FOXM1 overexpressing cells promoted the proliferation, migration and tube formation of HUVECs (Fig. 4a-c and Additional file 1: Figure S2A-B). Moreover, FOXM1 overexpressing xenografts showed higher microvessel density (MVD), determined by CD31 immunostaining, than control tumors (Fig. 4d and Additional file 1: Figure S2C). Consistently, knockdown of FOXM1 showed inhibitory effect on HUVECs (Additional file 1: Figure S2D-2G). To demonstrate whether miR-6868-5p inhibited CRC angiogenesis through targeting FOXM1, we performed rescue assays by using a vector expressing FOXM1 without its 3’-UTR, which avoided the miR-6868-5p-mediated suppression (Fig. 4e). Overexpression of FOXM1 could reverse the miR-6868-5p-induced inhibition of HUVECs proliferation and migration (Fig. 4f and g). Moreover, ectopic expression of FOXM1 counteracted the inhibition of endothelial tube formation caused by overexpression of miR-6868-5p (Fig. 4h). Together, these data confirmed our hypothesis that miR-6868-5p inhibited angiogenesis by targeting FOXM1 in CRC cells.

### miR-6868-5p/FOXM1 axis regulates CRC angiogenesis via IL-8

**Fig. 5:**
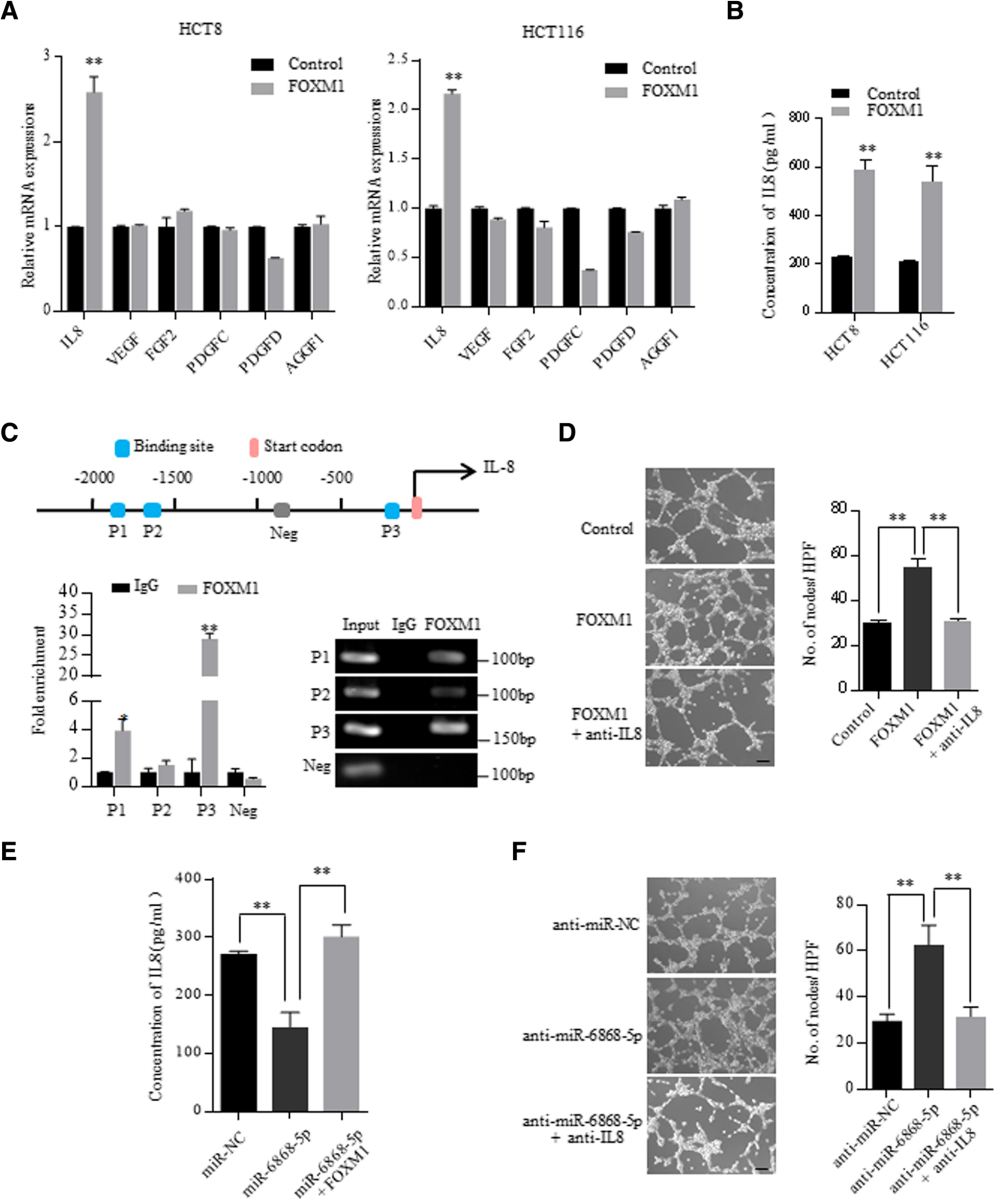
miR-6868-5p/FOXM1 axis regulates CRC angiogenesis via IL-8. **a** qRT-PCR analysis of the expression of angiogenic factors in HCT8 and HCT116 cells transfected with control vector or FOXM1 expressing plasmid. **b** IL-8 levels in the CM of indicated cells were determined by ELISA. **c** Upper: putative FOXM1 binding sites on the promoter region of IL-8. Lower: ChIP assay of the enrichment of FOXM1 on IL-8 promoter relative to IgG in HCT116 cells overexpressing FOXM1. A random region without FHREs served as a negative control (Neg). **d** Tube formation assay of HUVECs in response to CM from FOXM1 overexpressing HCT116 cells with or without anti-IL-8 neutralizing antibody (5 μg/mL) treatment. Scale bar = 20 μm. **e** IL-8 levels in the CM of indicated cells were determined by ELISA. **f** Tube formation assay of HUVECs in response to CM from miR-6868-5p inhibiting HCT116 cells with or without anti-IL-8 neutralizing antibody (5 μg/mL) treatment. Scale bar = 20 μm. *p < 0.05, **p < 0.01

FOXM1 has been reported to regulate tumor angiogenesis through promoting the transcription of angiogenic factors [16, 17]. To identify the angiogenic factors responsible for the miR-6868-5p/FOXM1 axis-regulated angiogenesis, we screened the promoter regions of angiogenic factors for FOXM1 binding sites. Six angiogenic factors with putative FOXM1 binding sites at the promoter region were selected out and subjected to qRT-PCR validation. As shown in Fig. 5a, the mRNA levels of IL-8 exhibited the most robust increase following FOXM1 overexpression. Pearson’s correlation analysis showed positive correlation between FOXM1 and IL-8 levels in CRC specimens from GEO datasets (Additional file 1: Figure S3). ELISA further confirmed the elevation of IL-8 in the CM of FOXM1 overexpressing CRC cells (Fig. 5b). Bioinformatic analysis identified three putative fork head response elements (FHREs) in the promoter region of IL-8 (Fig. 5c). Chromatin immunoprecipitation (ChIP) assay corroborated that FOXM1 was enriched on two FHREs of IL-8 promoter (Fig. 5c). Moreover, IL-8 neutralizing antibody treatment efficiently reversed FOXM1-enhanced endothelial tube formation (Fig. 5d). These results indicated that FOXM1 promoted tumor angiogenesis through activating IL-8 transcription.

We next investigated whether IL-8 was involved in miR-6868-5p/FOXM1- regulated angiogenesis. Overexpression of miR-6868-5p reduced the production of IL-8 in the CM (Fig. 5e). As expected, overexpression of FOXM1 could restore the IL-8 level (Fig. 5e). Furthermore, HUVECs cultured in CM from CRC cells transfected with miR-6868-5p inhibitor displayed higher tube formation ability than those cultured in the control group, whereas IL-8 neutralizing antibody treatment reversed the effect (Fig. 5f). These data implied that miR-6868-5p/FOXM1 axis regulates CRC angiogenesis via IL-8.

### Delivery of miR-6868-5p mimic suppresses tumor angiogenesis in vivo

**Fig. 6:**
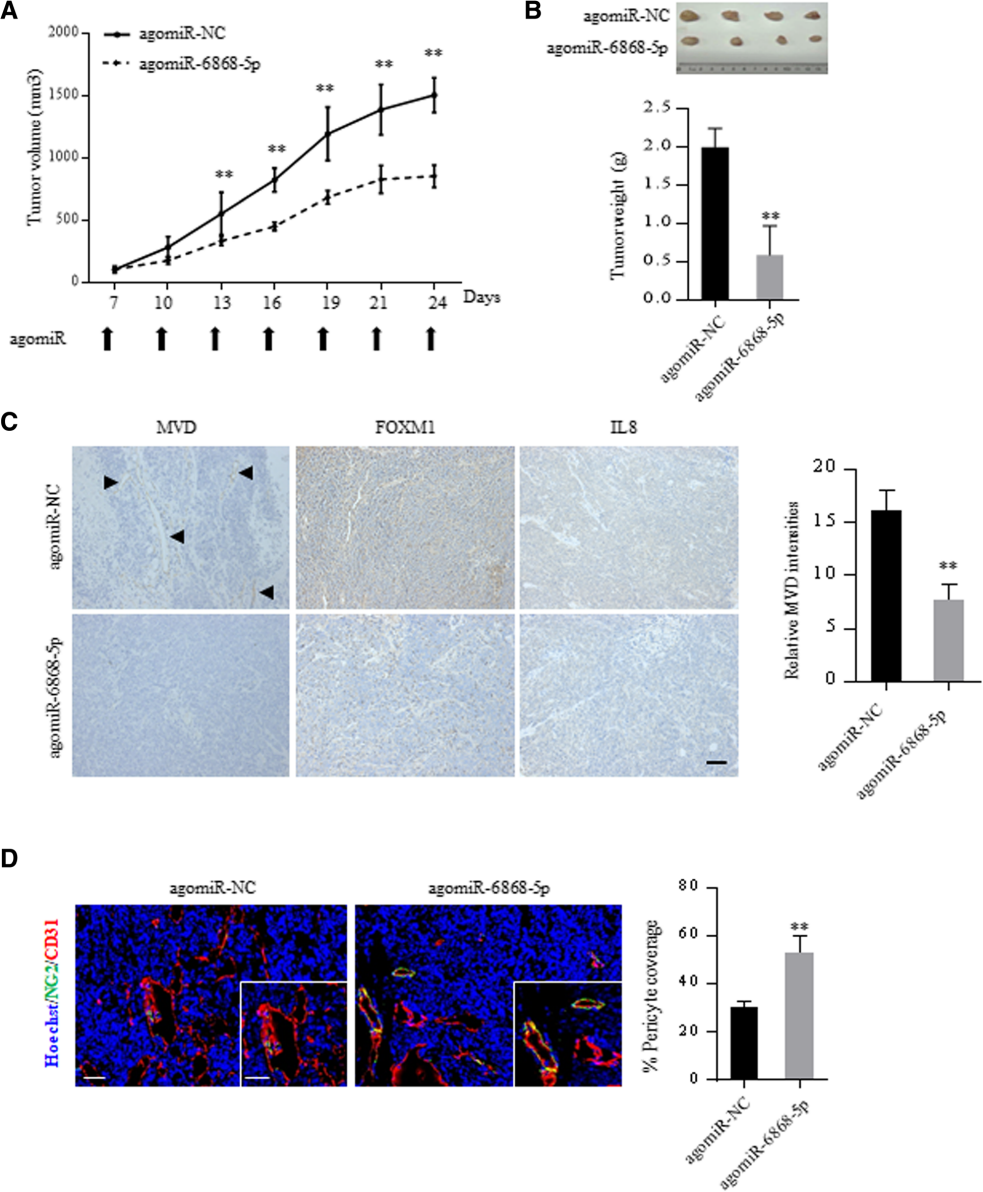
Delivery of miR-6868-5p mimic suppresses tumor angiogenesis in vivo. **a** The nude mice inoculated with HCT116 cells were divided into two treatment groups: control and miR-6868-5p agomir. Tumor size was measured every three days after inoculation. **b** Xenografted tumors were excised at week 4 post the inoculation, and the tumor weight from different groups was compared. **c** Representative images of IHC staining for CD31, FOXM1 and IL-8 in indicated agomir-treated tumors. Scale bar = 20 μm. **d** Representative images of CD31 (red), NG2 (green) and nuclei (blue) immunofluorescence staining in indicated agomir-treated tumors. Higher magnification images are shown in the insets. Scale bar = 50 μm. *p < 0.05, **p < 0.01

Given the critical role of miR-6868-5p in CRC angiogenesis, we explored the therapeutic efficacy of miR-6868-5p using mouse models. miR-6868-5p agomir or miR-NC agomir were injected intra-tumorally into nude mice bearing established subcutaneous tumor xenografts, and the effect on tumor growth was monitored. Delivery of miR-6868-5p agomir resulted in a significant decrease in tumor growth compared to treatment with miR-NC agomir (Fig. 6a and b). Histologic comparison of the tumor xenografts demonstrated lower MVD in the miR-6868-5p agomir-treated tumors compared with control tumors (Fig. 6c). In parallel, miR-6868-5p agomir treatment reduced FOXM1 and IL-8 expression in tumors (Fig. 6c). Colocalization analysis of CD31^+^ endothelial cells and NG2 ^+^pericytes showed a significant reduction in MVD, with an increase in pericytes upon miR-6868-5p overexpression (Fig. 6d), indicating impaired angiogenesis. Together, these results further demonstrated that miR-6868-5p is a potent inhibitor of CRC angiogenesis.

### FOXM1 inhibits miR-6868-5p transcription by promoter histone methylation via EZH2

**Fig. 7:**
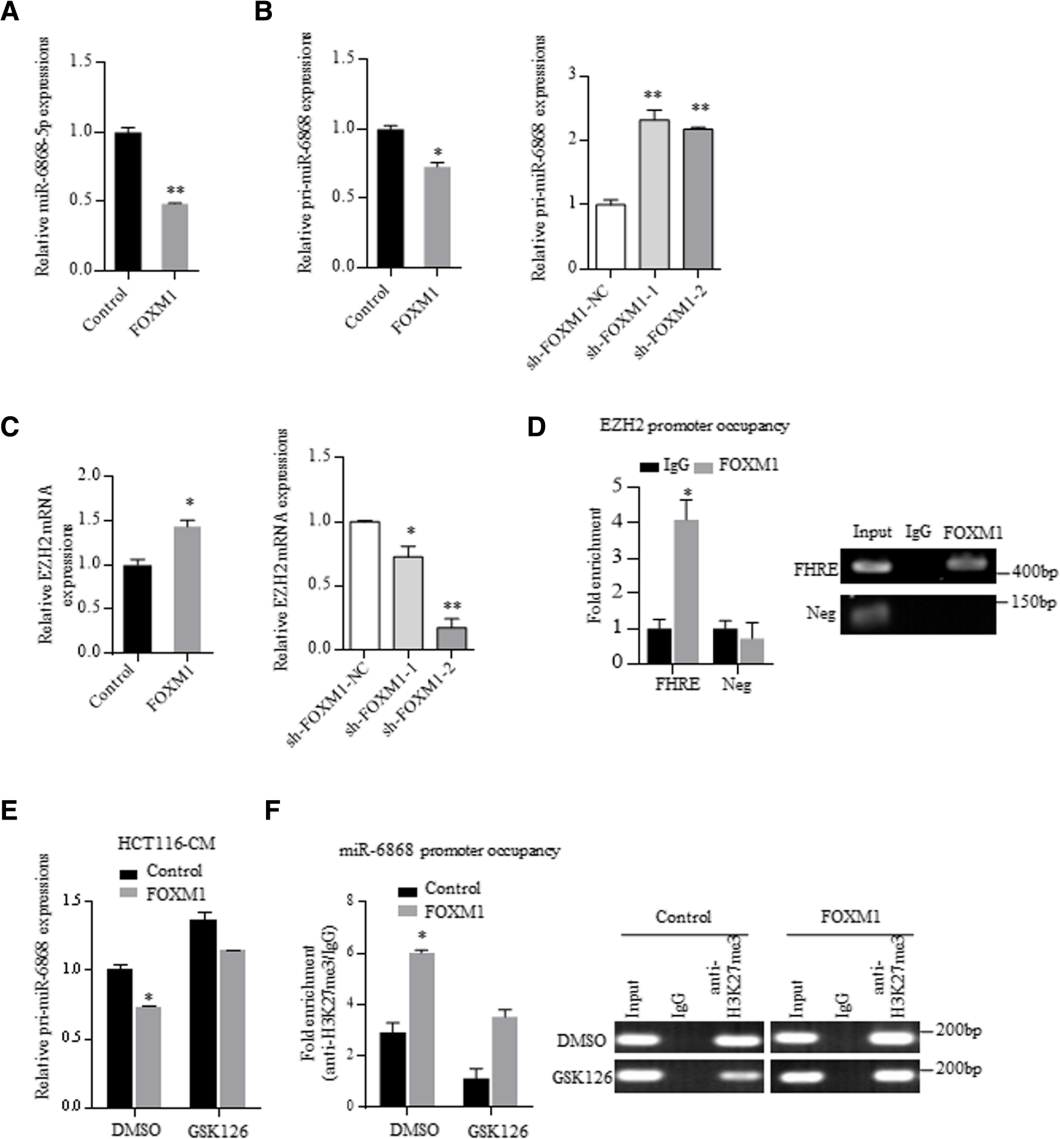
FOXM1 inhibits miR-6868-5p transcription by promoter histone methylation via EZH2. **a** qRT-PCR analysis of miR-6868-5p expression in HCT116 cells transfected with control vector or FOXM1 expressing plasmid. **b** qRT-PCR analysis of pri-miR-6868 expression in HCT116 cells with FOXM1 overexpressing or knockdown. **c** qRT-PCR analysis of EZH2 expression in HCT116 cells with FOXM1 overexpressing or knockdown. **d** ChIP assay of the enrichment of FOXM1 on EZH2 promoter relative to IgG in HCT116 cells. A random region without FHREs served as a negative control (Neg). **e** HCT116 cells transfected with control vector or FOXM1-expressing plasmid were treated with GSK126 (5 μM). The pri-miR-6868 levels were examined by qRT-PCR after 48 h. **f** ChIP assay of the enrichment of H3K27me3 on miR-6868 promoter relative to IgG in indicated HCT116 cells. *p < 0.05, **p < 0.01

Regulatory feedback loops have been reported between miRNAs and target genes [18, 19]. Intriguingly, we found that overexpression of FOXM1 downregulated miR-6868-5p levels (Fig. 7a). To determine how FOXM1 affected miR-6868-5p expression, we detected the expression levels of pri-miR-6868 and pre-miR-6868. Upregulation of FOXM1 resulted in a significant decrease of pri-miR-6868 levels (Fig. 7b), as well as pre-miR-6868 levels (Additional file 1: Figure S4A), indicating that FOXM1 inhibited the transcription of miR-6868. However, FOXM1 had no interaction with the promoter of miR-6868 (data not show), suggesting that FOXM1 did not directly suppress the transcription of miR-6868.

Epigenetic regulation, including DNA methylation and histone modifications, play an important role in gene expression regulation [20–22], thus we investigated whether epigenetic regulation was involved in FOXM1-inhibited miR-6868-5p expression. 5-Aza-CdR, an inhibitor of DNA methyltransferases (DNMTs), had no effect on miR-6868-5p level (Additional file 1: Figure S4B), excluding the involvement of DNA methylation in miR-6868-5p downregulation. Recently, FOXM1 has been reported to trans-activate EZH2 [23], which functions as a transcription repressor by catalyzing trimethylation of histone 3 at lysine 27 (H3K27me3) [24, 25], thus we speculated whether EZH2 was involved in FOXM1-elicited suppression of miR-6868-5p. In line with previous study, FOXM1 promoted the expression of EZH2 in CRC cells (Fig. 7c), and FOXM1 was enriched on the promoter of EZH2 (Fig. 7d). To determine the role of EZH2 in the regulation of miR-6868-5p expression, we treated FOXM1 overexpressing CRC cells with GSK126, a specific EZH2 inhibitor (Additional file 1: Figure S4C). As shown in Fig. 7e, GSK126 treatment could overturn the FOXM1-induced downregulation of pri-miR-6868. Furthermore, ChIP assay showed gain of H3K27me3 at the promoter of miR-6868 in FOXM1 overexpressing cells compared with control cells (Fig. 7f). However, GSK126 exposure reversed the enrichment of H3K27me3 at the promoter of miR-6868 in FOXM1 overexpression cells (Fig. 7f). These data indicated that FOXM1 led to EZH2-mediated H3K27me3 on miR-6868 promoter and suppressed pri-miR-6868 transcription, therefore forming a feedback circuit between miR-6868-5p and FOXM1 in CRC cells.

### miR-6868-5p is downregulated in human CRC and correlates with angiogenesis

**Fig. 8:**
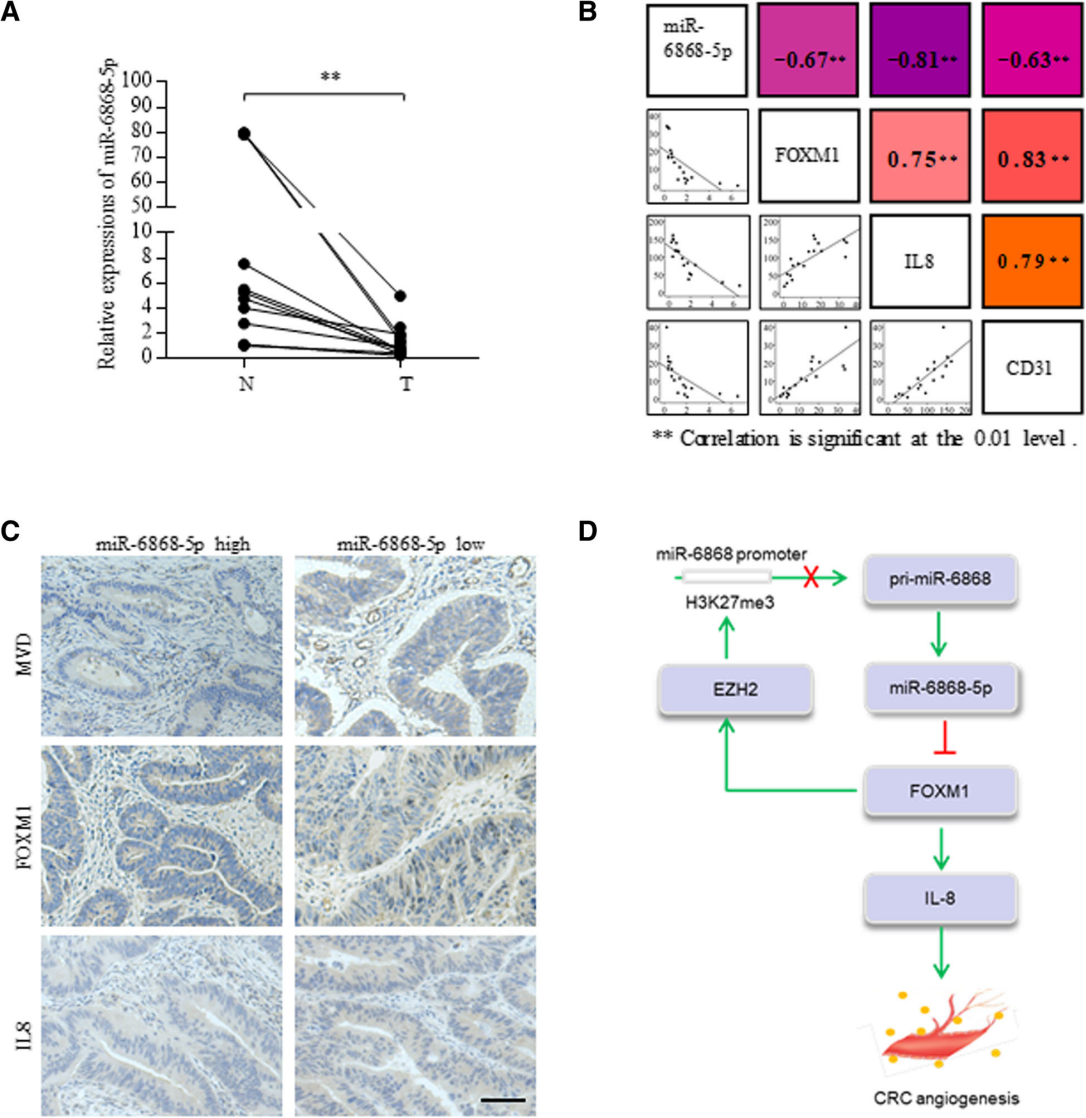
miR-6868-5p is downregulated in human CRC and correlates with angiogenesis. **a** qRT-PCR was used to quantify expression of miR-6868-5p in 14 pairs of CRC tissues and adjacent normal tissues. **b** The shaded squares in the upper right showed Spearman correlation values between the expression of indicated genes in CRC tissues. The lower left squares show the scatter plot and fitted trend lines for the same comparisons. ** Correlation is significant at the 0.01 level. **c** Representative view of immunostaining of CD31, FOXM1 and IL-8 in CRC tissue sections with high or low miR-6868-5p expression. Scale bars = 20 μm. **d** Schematic representation of the mechanism of miR-6868-5p/FOXM1circuit in the regulation of CRC angiogenesis

Lastly, to determine the clinical significance of miR-6868-5p, we examined the RNA levels of miR-6868-5p in human CRC tissues and their adjacent noncancerous tissues. Compared with adjacent normal tissues, miR-6868-5p expression was decreased in CRC samples (Fig. 8a). To demonstrate the impact of miR-6868-5p on angiogenesis in clinical samples, patient tumors were used to assess the correlation between miR-6868-5p expression and CD31 staining. As shown in Fig. 8b, linear correlation analysis showed that miR-6868-5p expression was inversely correlated with MVD, FOXM1 and IL-8 staining in CRC tissues. MiR-6868-5p expression was also negatively correlated with TNM stage (Additional file 1: Table S4). Immunochemistry staining of CRC sections showed that tumors with high levels of miR-6868-5p exhibited reduced expression of CD31, FOXM1 and IL-8 compared with those with low levels of miR-6868-5p (Fig. 8c). Collectively, our study demonstrated a miR-6868-5p/FOXM1 feedback loop in the manipulation of CRC angiogenesis.

## Discussion

FOXM1 has been reported as a master regulator in CRC, and can be used as a prognostic indicator for poor outcome [4, 26–29]. However, the factors that cause dysregulation of FOXM1 in CRC remain elusive. Our study focused on finding potential miRNAs that regulate FOXM1. Through an integrated analysis of software prediction, molecular and functional studies, we identified FOXM1 as a direct downstream target of miR-6868-5p and demonstrated the critical role of miR-6868-5p /FOXM1 axis in the regulation of tumor angiogenesis. To our knowledge, this study reports, for the first time, the role and mechanism of miR-6868-5p.

Angiogenesis is regulated by angiogenic factors [30, 31]. FOXM1 was shown to stimulate angiogenesis in several cancers, including pancreatic cancer, gastric cancer and glioma, through induction of vascular endothelial growth factor (VEGF), matrix metalloproteinase-2 (MMP-2) and MMP-9 [13–15]. In this study, we identified pro-angiogenic factor IL-8 as a novel transcriptional target of FOXM1, and showed that miR-6868-5p reduced angiogenesis and IL-8 expression by inhibiting FOXM1 expression. No conserved miR-6868-5p binding sites were identified in the 3’-UTR region of IL-8, excluding that IL-8 was directly targeted by miR-6868-5p, IL-8, also known as CXCL8, has been well documented to promote tumor angiogenesis mainly by binding to its receptor CXCR2 [32–34]. Targeted therapies against IL-8/CXCR2 axis are being tested in clinical trials for tumor treatment (eg. NCT02001974, NCT02370238, NCT02536469). For instance, a phase Ib pilot study is undergoing to assess HuMax-CXCL8, a CXCL8 neutralising antibody, in patients with metastatic or unresectable, locally advanced malignant solid tumors.

Recent advancement in miRNA-based therapy and delivery strategy have made it a promising way for cancer treatment [35–39]. In this case, it is essential to identify critical miRNAs that are associated with tumor progression. Given the important role of miR-6868-5p in tumor angiogenesis, we investigated its therapeutic potential in our study. Through intra-tumoral injection of modified agomir, we showed that miR-6868-5p can mediate potent anti-angiogenic and anti-tumor effects in CRC xenograft mouse model, suggesting that delivering miR-6868-5p to tumors holds great promise as therapeutic approach. For clinical application, delivering miRNAs systemically using potential carriers, such as nanoparticles or cell-derived membrane vesicles, is more feasible. The effect of systemic delivery of miR-6868-5p in cancer treatment remains to be established in future work.

Accumulating evidence established that regulatory feedback loops are substantial mechanisms underlying tumor biology. In this study, we found that FOXM1 downregulated the expression of miR-6868-5p, forming a feedback circuit between miR-6868-5p and FOXM1 in CRC cells. Noticeably, genome location of miR-6868 is inside an intron of EXOC7, proposing possibility that mature miR-6868 may be derived from the intronic splicing of EXOC7, other than Drosha-dependent canonical miRNA processing [40]. However, miR-6868-5p expression was not correlated with EXOC7 expression (Additional file 1: Figure S5A). Moreover, knockdown of Drosha led to the downregulation of pre-miR-6868 and miR-6868-5p (Additional file 1: Figure S5B), indicating that the biogenesis of miR-6868-5p was dependent on Drosha, but not the splicing of EXOC7. Our data also showed that FOXM1 had no effect on EXOC7 expression, therefore FOXM1 elicited suppression on miR-6868 was not attribute to the regulation of EXOC7 (Additional file 1: Figure S5C). EZH2, the catalytic subunit of polycomb repressive complex 2 (PRC2), mediates transcriptional repression by catalyzing trimethylation of H3K27. Our results demonstrated that FOXM1 promoted the expression of EZH2, which suppressed the transcription of miR-6868-5p via enhancing the H3K27me3 level at the promoter of miR-6868. Mutation or overexpression of EZH2 has been linked to many forms of cancer [41–44]. A multicenter, open-label, Phase 2 study (NCT03456726) to assess the efficacy and safety of tazemetostat, an EZH2 inhibitor, in participants with relapsed or refractory B-cell non-Hodgkin’s lymphoma is currently ongoing.

## Conclusions

Our results identified a feedback loop of miR-6868-5p/FOXM1, in which miR-6868-5p inhibits tumor angiogenesis by suppressing FOXM1-IL-8 axis, and in turn FOXM1 downregulates miR-6868-5p by stimulating EZH2-mediated transcriptional suppression of miR-6868-5p (Fig. 8d). Our study shed new light on the mechanisms of CRC angiogenesis and provide candidate therapeutic targets for the management of CRC.

## Additional file

Additional file 1: **Figure S1.** (A) qRT-PCR analysis of the expression of indicated miRNAs in HCT8 and HCT116 cells. (B & C) Transfection efficiency was measured by qRT-PCR. *p < 0.05, **p < 0.01. **Figure S2.** (A) Western blot analysis of FOXM1 expression in HCT116 cells transfected with empty control vector or FOXM1 expressing plasmid. (B) HUVECswere treatedwith the CM from indicated cells, and subjected to wound healing assay. Scale bar = 20 μm. (C) Xenografted tumors were excised at week 3 post the inoculation, and the tumor weight from different groups was compared. (D) Western blot analysis of FOXM1 expression in HCT116 cells transfected with shNC, sh1 and sh2 against FOXM1. (E) HUVECs were treated with the CM from HCT116 cells transfected with indicated vectors. Cell viability of HUVECs was measured by CCK8 assay. (F) HUVECs were co-cultured with HCT116 cells transfected with indicated vectors in transwell apparatus. Migrated HUVECs were quantified after co-culture for 24 h. Scale bar = 20 μm. (G) HUVECs were treated with the CM from indicated cells, and subjected to tube formation assay. Scale bar = 20 μm. *p < 0.05, **p < 0.01. **Figure S3.** The bivariate relation between the mRNA levels of FOXM1 and IL-8 in CRC samples from GEO dataset was assessed by Pearson’s correlation test. **Figure S4.** (A) qRT-PCR analysis of pre-miR-6868 expression in HCT116 cells with FOXM1 overexpression. (B) HCT8 and HCT116 cells were treated with 5-Azd (10 μM). The miR-6868-5p levels were examined by qRT-PCR after 48 h. miR-375 was used as positive control. (C) Western blot analysis of H3K27me3 expression in HCT116 cells upon GSK126 treatment. *p < 0.05, **p < 0.01. **Figure S5.** (A) The bivariate relation between the EXOC7 mRNA levels and miR-6868-5p levels in CRC samples was assessed by Pearson’s correlation test. (B) qRT-PCR analysis of pre-miR-6868 and miR-6868-5p expression in cells transfected with siNC or siDrosha. (C) qRT-PCR analysis of EXOC7 expression in HCT116 cells with FOXM1 overexpression. **Table S1.** Number of predicted binding sites in FOXM1 3’-UTR. **Table S2.** Sequences of primers used for qRT-PCR in this study. **Table S3.** Sequences of primers used for ChIP-qPCR in this study. **Table S4.** Correlation between miR-6868-5p expression and TNM stage in CRC samples. (DOCX 12322 kb)

## Abbreviations

ChIP: Chromatin immunoprecipitation
CM: Conditioned medium
CRC: Colorectal cancer
FHREs: Fork head response elements
FOXM1: Transcription factor forkhead box M1
MVD: Microvessel density
