# Supplementary material for: Dysregulation of miR-6868-5p/FOXM1 circuit contributes to colorectal cancer angiogenesis
Source: J Exp Clin Cancer Res. 2018 Nov 28;37:292. doi: 10.1186/s13046-018-0970-5 (PMC6264626; doi:10.1186/s13046-018-0970-5)
Supplement: Supplementary file 1 — Figure S1. (A) qRT-PCR analysis of the expression of indicated miRNAs in HCT8 and HCT116 cells. (B & C) Transfection efficiency was measured by qRT-PCR. *p < 0.05, **p < 0.01. Figure S2. (A) Western blot analysis of FOXM1 expression in HCT116 cells transfected with empty control vector or FOXM1 expressing plasmid. (B) HUVECswere treatedwith the CM from indicated cells, and subjected to wound healing assay. Scale bar = 20 μm. (C) Xenografted tumors were excised at week 3 post the inoculation, and the tumor weight from different groups was compared. (D) Western blot analysis of FOXM1 expression in HCT116 cells transfected with shNC, sh1 and sh2 against FOXM1. (E) HUVECs were treated with the CM from HCT116 cells transfected with indicated vectors. Cell viability of HUVECs was measured by CCK8 assay. (F) HUVECs were co-cultured with HCT116 cells transfected with indicated vectors in transwell apparatus. Migrated HUVECs were quantified after co-culture for 24 h. Scale bar = 20 μm. (G) HUVECs were treated with the CM from indicated cells, and subjected to tube formation assay. Scale bar = 20 μm. *p < 0.05, **p < 0.01. Figure S3. The bivariate relation between the mRNA levels of FOXM1 and IL-8 in CRC samples from GEO dataset was assessed by Pearson’s correlation test. Figure S4. (A) qRT-PCR analysis of pre-miR-6868 expression in HCT116 cells with FOXM1 overexpression. (B) HCT8 and HCT116 cells were treated with 5-Azd (10 μM). The miR-6868-5p levels were examined by qRT-PCR after 48 h. miR-375 was used as positive control. (C) Western blot analysis of H3K27me3 expression in HCT116 cells upon GSK126 treatment. *p < 0.05, **p < 0.01. Figure S5. (A) The bivariate relation between the EXOC7 mRNA levels and miR-6868-5p levels in CRC samples was assessed by Pearson’s correlation test. (B) qRT-PCR analysis of pre-miR-6868 and miR-6868-5p expression in cells transfected with siNC or siDrosha. (C) qRT-PCR analysis of EXOC7 expression in HCT116 cells with FOXM1 overexpression. Table [file 13046_2018_970_MOESM1_ESM.docx]

**Figure S1.** (A) qRT-PCR analysis of indicated miRNAs expression in HCT8 and HCT116 cells. (B & C) Transfection efficiency was measured by qRT-PCR. **Figure S2.** (A) Western blot analysis of FOXM1 expression in control and FOXM1 overexpressing HCT116 cells. (B) HUVECs were treated with the CM from indicated cells, and subjected to wound healing assay. Scale bar = 20 µm. (C) The weight of xenografted tumors from different groups was compared. (D) Western blot analysis of FOXM1 expression in HCT116 cells transfected with shNC, sh1 and sh2 against FOXM1. (E) HUVECs were treated with the CM from indicated cells, and subjected to CCK8 assay. (F) HUVECs were co-cultured with HCT116 cells transfected with indicated vectors in transwell apparatus. Migrated HUVECs were quantified after co-culture for 24h. Scale bar = 20 µm. (G) HUVECs were treated with the CM from indicated cells, and subjected to tube formation assay. Scale bar = 20 µm. **Figure S3.** The bivariate relation between FOXM1 and IL-8 levels in CRC samples from GEO dataset. **Figure S4.** (A) qRT-PCR analysis of pre-miR-6868 expression in HCT116 cells with FOXM1 overexpression. (B) HCT8 and HCT116 cells were treated with 5-Azd (10µM). The miR-6868-5p levels were examined by qRT-PCR after 48h. miR-375 was used as positive control. (C) Western blot analysis of H3K27me3 expression in HCT116 cells upon GSK126 treatment. **Figure S5.** (A) The bivariate relation between the EXOC7 and miR-6868-5p levels in CRC samples. (B) qRT-PCR analysis of pre-miR-6868 and miR-6868-5p expression in cells transfected with siNC or siDrosha. (C) qRT-PCR analysis of EXOC7 expression in HCT116 cells with FOXM1 overexpression. **Table S1**. Number of predicted binding sites in FOXM1 3’-UTR. **Table S2.** Sequences of primers used for qRT-PCR. **Table S3.** Sequences of primers used for ChIP-qPCR. **Table S4**. Correlation between miR-6868-5p expression and TNM stage in CRC samples.
